# MEMS piezoelectric resonant microphone array for lung sound classification

**DOI:** 10.1088/1361-6439/acbfc3

**Published:** 2023-03-09

**Authors:** Hai Liu, Matin Barekatain, Akash Roy, Song Liu, Yunqi Cao, Yongkui Tang, Anton Shkel, Eun Sok Kim

**Affiliations:** 1 Department of Electrical and Computer Engineering, University of Southern California, Los Angeles, CA, United States of America

**Keywords:** piezoelectric MEMS microphone, resonant microphone array, acoustic transducer, lung sounds classification, wearable health sensor

## Abstract

This paper reports a highly sensitive piezoelectric microelectromechanical systems (MEMS) resonant microphone array (RMA) for detection and classification of wheezing in lung sounds. The RMA is composed of eight width-stepped cantilever resonant microphones with Mel-distributed resonance frequencies from 230 to 630 Hz, the main frequency range of wheezing. At the resonance frequencies, the unamplified sensitivities of the microphones in the RMA are between 86 and 265 mV Pa^−1^, while the signal-to-noise ratios (SNRs) for 1 Pa sound pressure are between 86.6 and 98.0 dBA. Over 200–650 Hz, the unamplified sensitivities are between 35 and 265 mV Pa^−1^, while the SNRs are between 79 and 98 dBA. Wheezing feature in lung sounds recorded by the RMA is more distinguishable than that recorded by a reference microphone with traditional flat sensitivity, and thus, the automatic classification accuracy of wheezing is higher with the lung sounds recorded by the RMA than with those by the reference microphone, when tested with deep learning algorithms on computer or with simple machine learning algorithms on low-power wireless chip set for wearable applications.

## 1. Introduction

About 7.4% of the world population suffer from chronic respiratory diseases, among which asthma and chronic obstructive pulmonary disease (COPD) are most common [1, 2]. As many as 262 million people are affected by asthma and more than 1200 individuals die from asthma every single day on average [3]. Wheezing due to narrowing airway of the lung caused by asthma is a common symptom and can be sensed by a stethoscope [4–7]. Thus, lung sound monitoring can be very helpful for asthma patients, especially children, who cannot carry out the well-established pulmonary function tests accurately due to their inability to understand or follow the instruction on how to force air out of their lungs. Lung sounds can be monitored with electronic stethoscopes, but not for more than 1 h continuously due to their bulkiness and heaviness (stemming from the acoustic coupler needed to amplify faint lung sound) [8–10]. Also, a weak wheezing may be missed because of a low signal-to-noise ratio (SNR) of the microphone (used in the stethoscope). The published sensitivities of commercial MEMS condenser microphones are between 5 (TDK INMP411) [11] and 25.12 mV Pa^−1^ (TDK ICS-40730) [12], which depends on the applied bias voltage (typically about 10 V_DC_). Their SNRs for 1 Pa sound pressure are between 59 (Knowles SPH2430HR5H-B) [13] and 74 dBA (TDK ICS-40730) [12]. A MEMS condenser microphone with 22.39 mV Pa^−1^ sensitivity and 73 dBA SNR over 22 Hz–22 kHz has been reported, but with 200 V_DC_ applied bias voltage [14]. In the case of piezoelectric MEMS microphones, a bias voltage is not needed, and an unamplified sensitivity of 38 mV Pa^−1^ over 100–700 Hz with the fundamental resonance at 890 Hz has been reported [15].

Microphone sensitivity is enhanced at the mechanical resonance of a microphone diaphragm when the resonance’s quality factor (*Q*) is greater than 1, and MEMS resonant microphones have been reported [16–23]. An array of such resonant microphones can mimic the human auditory system based on resonances of 30 000 cochlear hairs at the basilar membrane [24, 25]. A higher *Q* means a higher sensitivity at the resonance frequency, but over a narrower bandwidth. Thus, a diaphragm with multiple resonances or a resonant microphone array (RMA) consisting of multiple resonant microphones covering different frequencies is needed to cover a wide frequency range. A piezoelectric RMA with unamplified sensitivities of the resonant microphones being 34.6–131.4 mV Pa^−1^ at their resonance frequencies between 169 and 662 Hz was reported for lung sound monitoring [21]. However, the noise floor and SNR of the RMA was not reported in [21].

This paper presents the design, fabrication, characterization, and application of a highly sensitive piezoelectric MEMS RMA for detection and automatic classification of wheezing in lung sounds. Measured unamplified sensitivity and SNR of the RMA are presented along with machine learning algorithms developed and implemented on a computer and on a commercial wireless chip set CYBLE-416045-02. Also presented are measured classification accuracies and speeds (directly related to energy consumption) for wheezing in lung sounds.

## 2. Design

**Figure 1.**
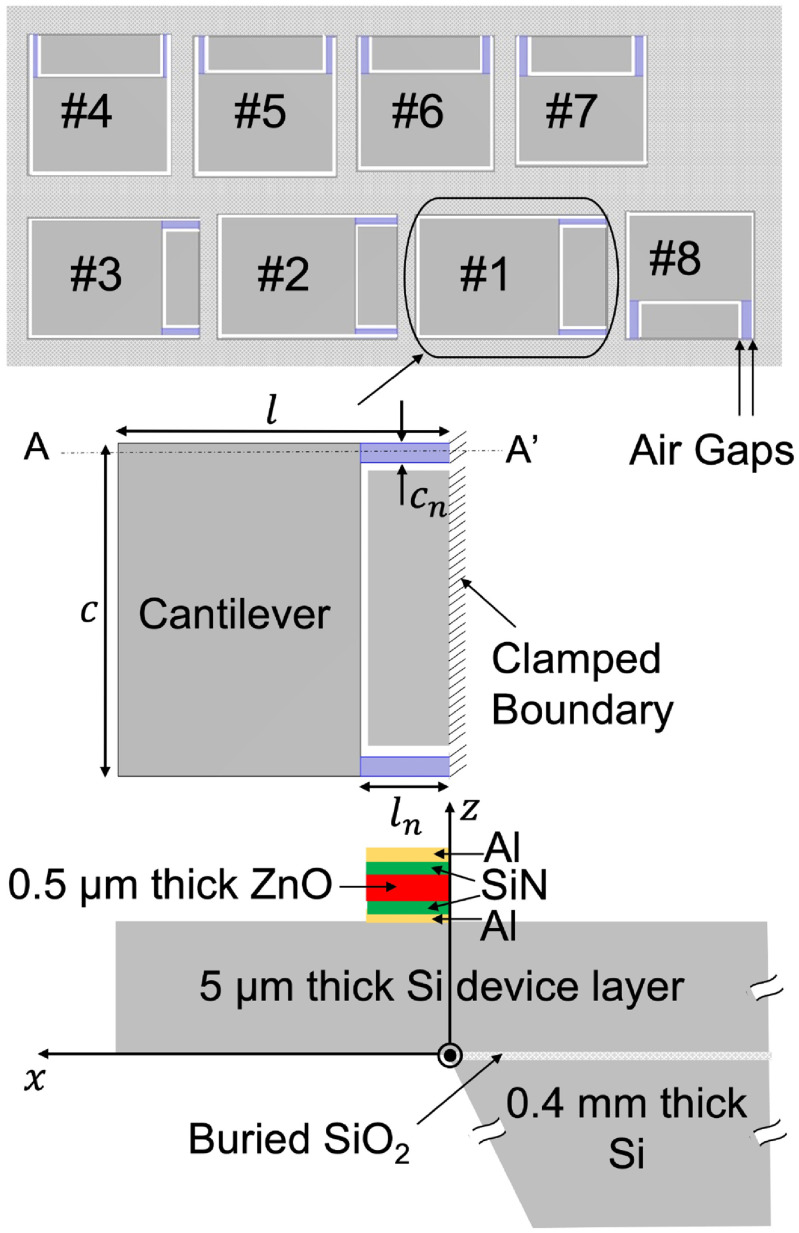
Resonant microphone array (RMA) schematic for lung sound classification with eight width-stepped cantilevers (*top*); top-view (*middle*) and cross-sectional view (*bottom*) of each width-stepped cantilever.

Eight of the width-stepped Si cantilevers, with two narrow beams supporting a rectangular plate, are used for the resonant microphones in the RMA (figure 1). The resonance frequencies are Mel-spaced (denser at lower frequencies as humans are capable of distinguishing lower frequencies better) between 200 and 800 Hz (frequency range of wheezing). More cantilevers can cover more frequencies with highly sensitive resonances, albeit at the cost of larger size for the RMA. For the wheezing detection over 200–800 Hz, eight cantilevers offer good trade-off between the performance and the size. Piezoelectric thin film ZnO, which converts the cantilever bending stress (due to applied sound pressure) to voltage, is placed only over the narrow support beams for maximum average stress over a largest possible area. Electrical insulation layer SiN encapsulates ZnO to prevent charge transfer between the top and bottom electrodes through ZnO for good sensitivity at low frequencies, as the resistivity of ZnO (${10^7}\,{{\Omega }} \cdot {\text{cm}}$) is relatively low. The air gap between the cantilever and the Si base is as narrow as 20 *μ*m to minimize acoustic pressure leakage at low frequencies. The sizes of the cantilever resonant microphones are 3.6–2.3 mm (table 1), while the thickness of the Si cantilever is 5 *μ*m (table 2).

**Table 1.** Resonance frequencies and sizes of the resonant microphones in the RMA in figure 1.

		Outline (mm)		Narrow part (mm)	
No.	Resonance frequency $f$	Length $l$	Width $c$	Length ${l_n}$	Width ${c_n}$
1	253 Hz	3.6	2.3	0.9	0.13
2	308 Hz	3.4	2.3	0.8	0.14
3	367 Hz	3.2	2.2	0.7	0.15
4	429 Hz	2.6	2.6	0.8	0.115
5	495 Hz	2.6	2.6	0.7	0.15
6	565 Hz	2.5	2.5	0.7	0.17
7	639 Hz	2.4	2.4	0.75	0.18
8	717 Hz	2.3	2.3	0.7	0.185

**Table 2.** The layer thicknesses of a width-stepped cantilever resonant microphone in figure 1.

Layer #	Name	Thickness $h$
1	Si device layer	5 $\mu {\text{m}}$
2	Al ground electrode	0.2 $\mu {\text{m}}$
3	SiN insulator	0.1 $\mu {\text{m}}$
4	Piezoelectric ZnO	0.5 $\mu {\text{m}}$
5	SiN insulator	0.1 $\mu {\text{m}}$
6	Al top electrode	0.2 $\mu {\text{m}}$

The fundamental resonance frequency of a width-stepped cantilever can be calculated through a beam free vibration equation for the cantilever displacements ${W_1}\left( x \right)$ and ${W_2}\,\left( x \right)$ (equation (1)) for the two parts having different widths

\begin{equation*}\left\{ {\begin{array}{*{20}{c}} {\frac{{{d^4}{W_1}\left( x \right)}}{{d{x^4}}} - \beta _1^4{W_1}\left( x \right) = 0,\,\,\,\,\,\,0 \leqslant x \leqslant {l_n}} \\[2pt] {\frac{{{d^4}{W_2}\left( x \right)}}{{d{x^4}}} - \beta _2^4{W_2}\left( x \right) = 0,\,\,\,\,\,\,{l_n} \lt x \leqslant l} \end{array}} \right.\end{equation*}

where $\beta _1^4 = \frac{{{m_1}{\omega ^2}}}{{{E_1}{I_1}}}\,{\textrm{and}}\,\beta _2^4 = \frac{{{m_2}{\omega ^2}}}{{{E_2}{I_2}}},\,$ with ${m_1}\,{\textrm{and}}\,{m_2}$ being the mass per unit length, ${E_1}\ {\textrm{and}}\ {E_2}$ being the Young’s modulus, and ${I_1}\,{\textrm{and}}\,\,\,{I_2}$ being the moment of inertia, at part 1 and 2, respectively. Once ${\beta _1}$ and ${\beta _2}$ are solved through equation (1) [26], the resonance frequency $f$ can be obtained as follows

\begin{equation*}f = \frac{{\beta _1^2}}{{2\pi }}\sqrt {\frac{{{E_1}{I_1}}}{{{m_1}}}} = \frac{{\beta _2^2}}{{2\pi }}\sqrt {\frac{{{E_2}{I_2}}}{{{m_2}}}} .\end{equation*}

**Figure 2.**
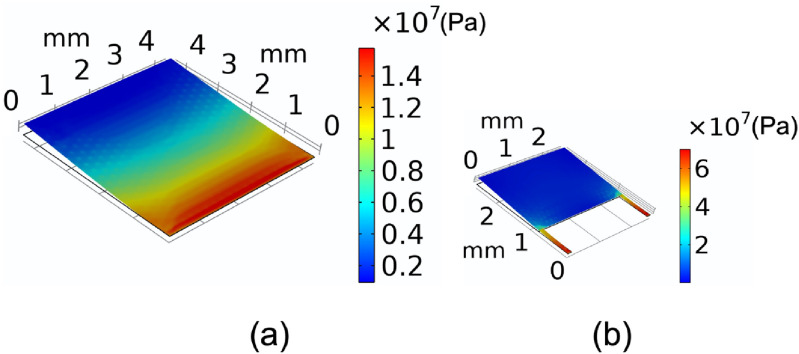
Finite-element-analysis (FEA) simulated stresses of $5\,\;\mu {\text{m }}$ thick cantilevers having the same fundamental resonant frequency of 436 Hz under 1 Pa pressure; (a) a standard cantilever of $4.35 \times 4.35\,\;{\text{m}}{{\text{m}}^2}$ showing $11\;{\text{MPa}}$ average stress near the fixed end and (b) a width-stepped cantilever of $2.6\;\, \times \;2.6\;{\text{m}}{{\text{m}}^2}$ with $57\,\;{\text{MPa}}$ average stress near the fixed ends of the two supporting beams.

**Figure 3.**
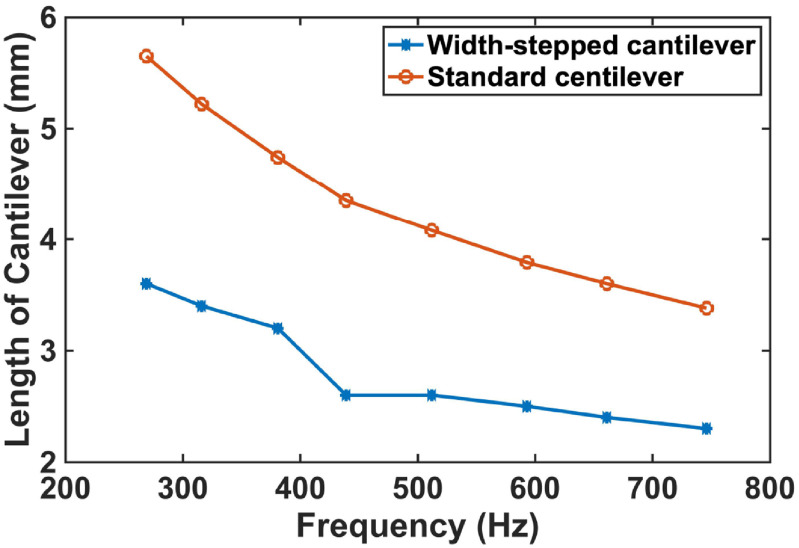
Calculated lengths of a width-stepped cantilever (table 1) and a standard cantilever vs fundamental resonance frequency.

**Figure 4.**
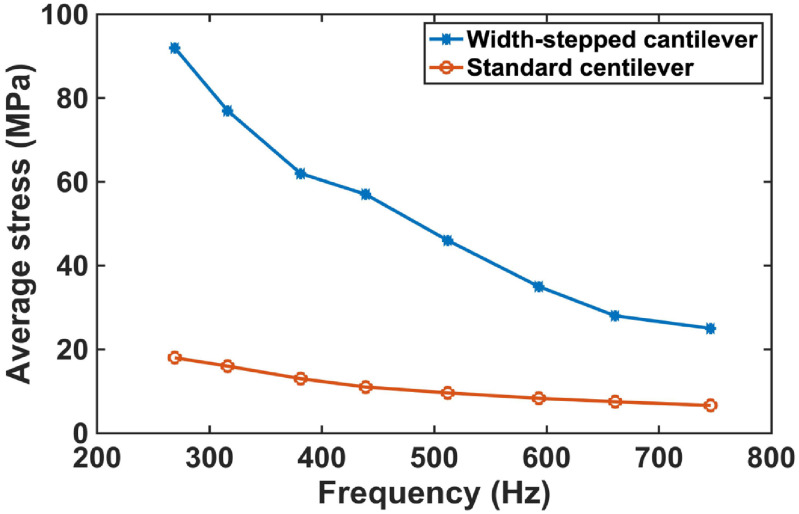
Calculated average stresses near the fixed ends for the width-stepped cantilever (table 1) and standard cantilever under 1 Pa sound pressure vs fundamental resonance frequency.

Compared to a rectangular cantilever with one fixed and three free ends (figure 2(a)), the bending-induced stress due to an applied pressure and the cantilever size for a same fundamental frequency are higher and smaller, respectively, for a width-stepped cantilever (figures 2–4). Thus, a higher unamplified sensitivity is expected with a piezoelectric microphone built on a width-stepped cantilever than that on a standard cantilever, as a piezoelectric film ZnO is placed only on the support beams of a width-stepped cantilever (figure 1). The voltage *V* produced across the ZnO thickness due to average stress *σ* induced by bending caused by an applied pressure is

\begin{equation*}V = \frac{{\sigma \times {d_{31}} \times A}}{C} = \frac{{\sigma \times {d_{31}} \times A}}{{\frac{{{\epsilon _0}{\epsilon _r}A}}{t}}} = \frac{{\sigma \times {d_{31}} \times t}}{{{\epsilon _0}{\epsilon _r}}}\end{equation*}

where $C,\,A\,\;{\text{and}}\,\;t$ are the ZnO’s capacitance, area, and thickness, respectively, while ${d_{31}}$ and ${\epsilon _r}$ are the piezoelectric coefficient and relative permittivity of the piezoelectric film, respectively, with ${\epsilon _0}$ being vacuum permittivity.

The resonance frequency of a width-stepped cantilever is not only dependent on the size of the whole cantilever but also on the size of the narrow segment as shown in table 1. Therefore, there is more design flexibility for the width-stepped cantilever. To make the RMA illustrated in figure 1 smaller, the width-stepped cantilever #4 (having the fundamental resonance frequency of 429 Hz) in the array is designed to have the same length $l$ as #5 (having the fundamental resonance frequency of 495 Hz) but with longer and narrower Narrow Part (table 1). This is why the curves of the length, average stress, and 2nd resonance frequency are not smooth for the width-stepped cantilever at #4 in figures 3, 4 and 6, respectively.

**Figure 5.**
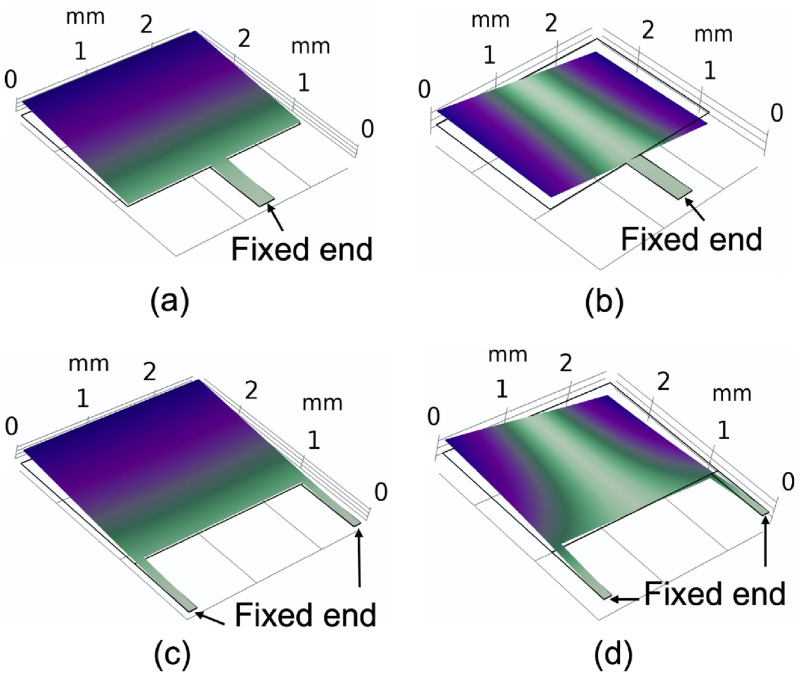
Simulated vibrations of width-stepped cantilevers at (a) and (b) the fundamental (436 Hz) and second-harmonic (949 Hz) resonance frequencies, respectively, with one narrow support beam in the center, (c) and (d) the fundamental (436 Hz) and second-harmonic (2049 Hz) resonance frequencies, respectively, with two narrow support beams at the two ends. The width of the one narrow beam in the center is twice that of the narrow beam at the end, while the length is the same. The total width and length of the cantilevers are the same to be $2.6\,\; \times \;2.6\,\;{\text{m}}{{\text{m}}^2}$.

**Figure 6.**
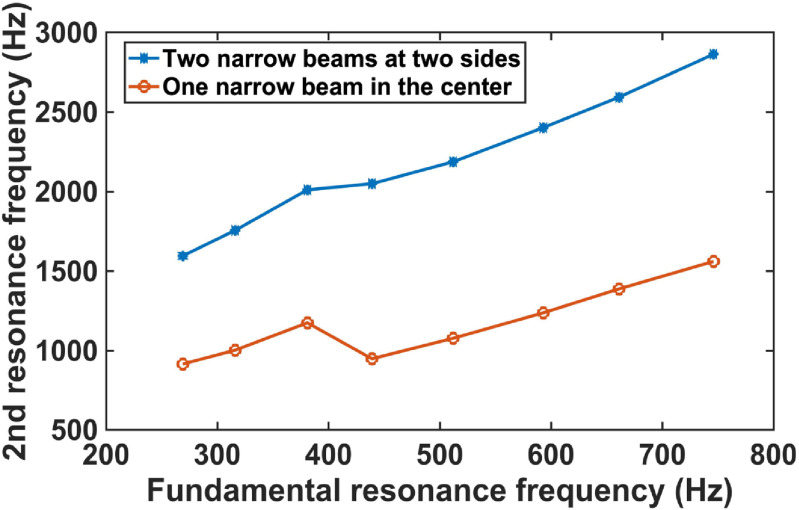
Simulated the 2nd resonance frequencies of the two types of width-stepped cantilevers vs fundamental resonance frequency. For each fundamental resonance frequency, the total length and width of the two types of the cantilevers are the same. The width of the one narrow beam in the center is twice of the beam width at the ends, while the length is the same.

A width-stepped cantilever with one narrow support beam in the center (figure 5(a)) has the 2nd harmonic resonance frequency close to the fundamental resonance frequency (figures 5(b) and 6), which may result in the 2nd resonance overlapping the fundamental resonance of another cantilever in an RMA. As we would like to utilize the fundamental resonance of each resonant microphone in an RMA and avoid any interference of the harmonics, a width-stepped cantilever with two narrow support beams (figures 5(c), (d) and 6) is designed for each resonant microphone in the RMA.

## 3. Fabrication

**Figure 7.**
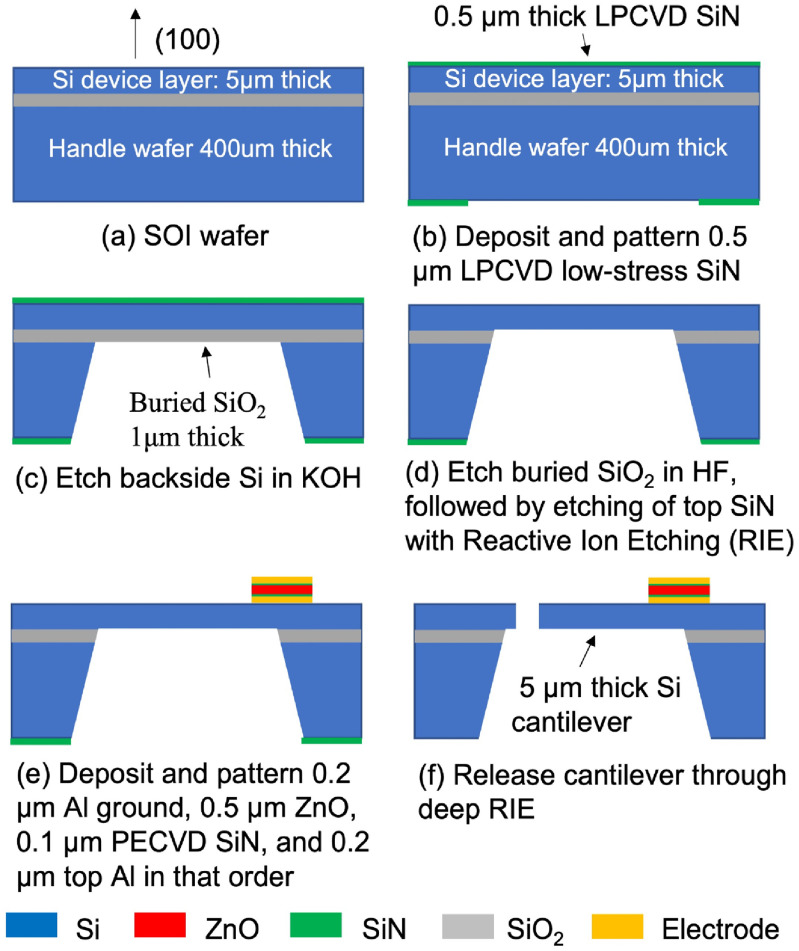
Brief fabrication process of the RMA.

The RMA is fabricated on a silicon-on-insulator wafer with 5 *μ*m thick Si device layer (figure 7(a)). First, 0.5 *μ*m thick low-stress SiN is deposited with low pressure chemical vapor deposition and patterned (figure 7(b)) for etch mask during KOH etching the Si (figure 7(c)). After etching the buried SiO_2_ in buffered HF, followed by etching of the top SiN in reactive ion etching (figure 7(d)), we sputter-deposit and pattern Al and piezoelectric ZnO, deposit SiN with plasma-enhanced chemical vapor deposition (PECVD) and pattern SiN, and then sputter-deposit and pattern Al (figure 7(e)). After dicing RMAs from the wafer, the cantilevers are released on each chip through etching Si on the diaphragms of each RMA (figure 7(f)).

**Figure 8.**
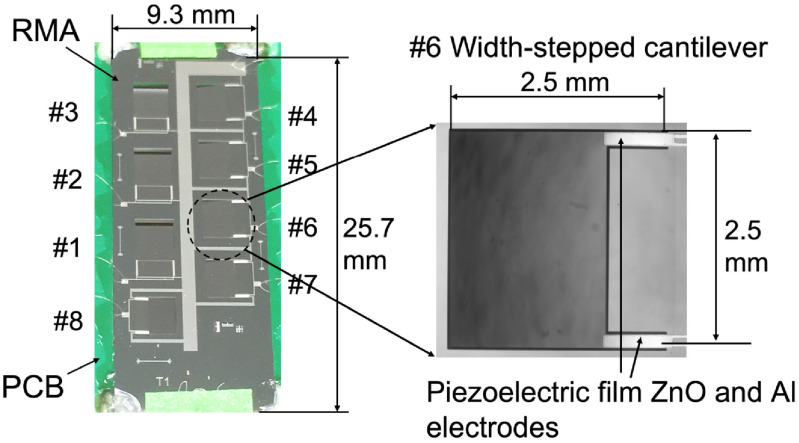
Photos of a fabricated RMA with eight width-stepped cantilever resonant microphones.

Long cantilevers (particularly, #1, #2 and #3) in the fabricated RMA (figure 8) show substantial downward warpage due to relatively large compressive residual stress in the ZnO film (table 3). The residual stresses of the thin films in table 3 are the average values calculated through measuring the curvature of a 3″ wafer by a profilometer DektakXT before and after the film deposition.

**Table 3.** Measured residual stresses of thin films in the cantilever.

Film	Stress (MPa)
Sputter-deposited ZnO	−350
Sputter-deposited Al	−30
PECVD SiN	−50

## 4. Characterization

### 4.1. Unamplified sensitivity

The measured capacitances and resistances of the resonant microphones (table 4) are close to the designed values.

**Table 4.** Measured paralleled capacitance and resistance of the resonant microphones in the RMA.

Microphone #	Capacitance	Resistance
1	23.9 pF	>10 GΩ
2	25.4 pF	>10 GΩ
3	23.5 pF	>10 GΩ
4	24 pF	>10 GΩ
5	26.3 pF	>10 GΩ
6	27.6 pF	>10 GΩ
7	28.5 pF	>10 GΩ
8	27.2 pF	>10 GΩ

**Figure 9.**
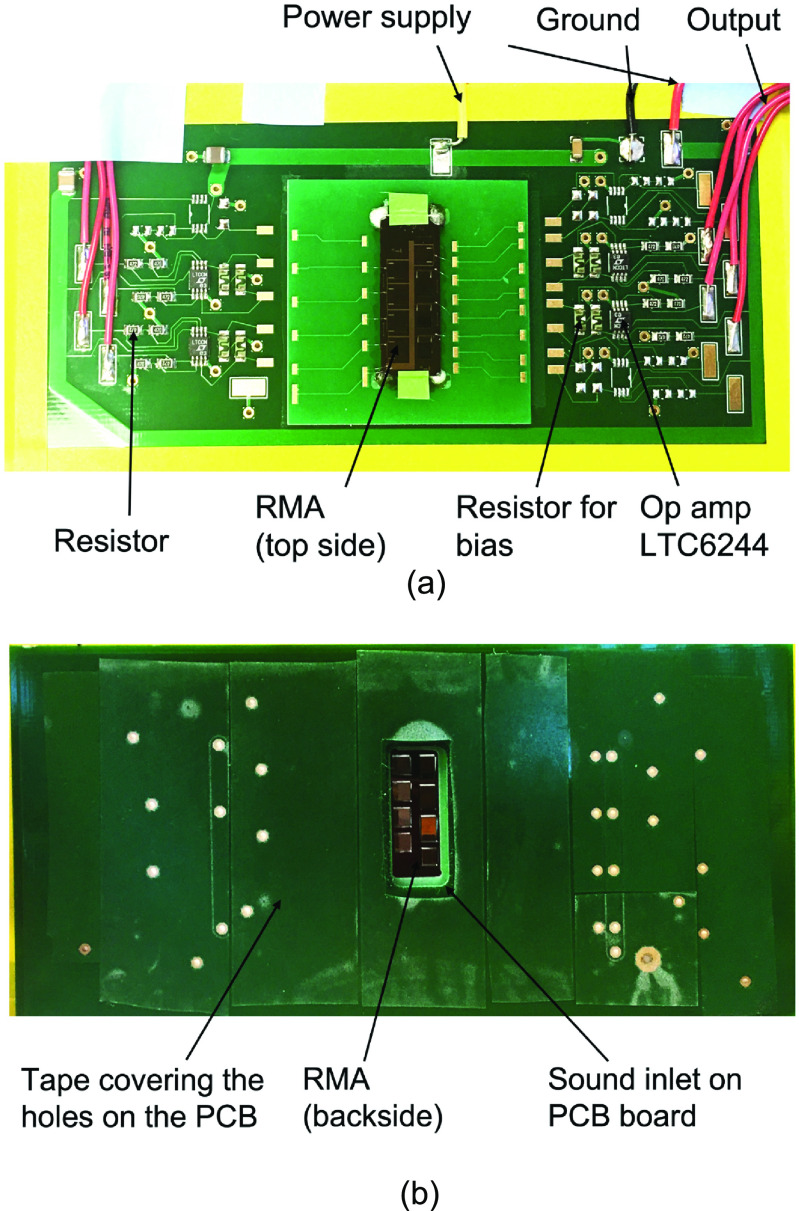
Top-side (a) and bottom-side (b) photos of the RMA on a printed circuit board (PCB) with op-amp based circuits.

**Figure 10.**
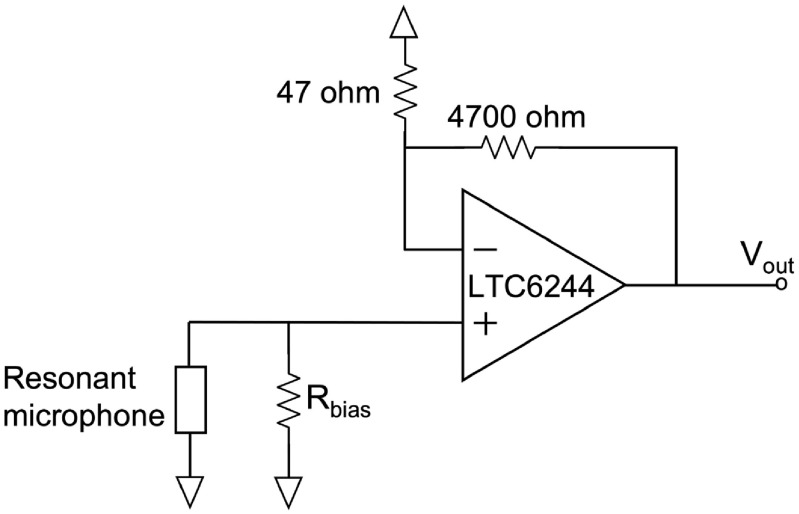
Schematic of non-inverting op-amp-based amplification circuit for each piezoelectric resonant microphone in the RMA.

The fabricated RMA is placed over a slot (for sound input) in a printed circuit board (PCB) (figure 9) and voltage amplifiers based on LTC6244 op amp with input resistance and capacitance of 10^12^ Ω and 2.1 pF, respectively (figure 10). The signal from each resonant microphone of the RMA is magnified and recorded separately without connecting to each other. The measured sensitivity from the amplifier output is divided by the amplification factor of 101 for unamplified sensitivity. A bias resistor of 1 GΩ (figure 10) is used for DC-biasing the op amp without affecting the low frequency response of the piezoelectric microphone.

**Figure 11.**
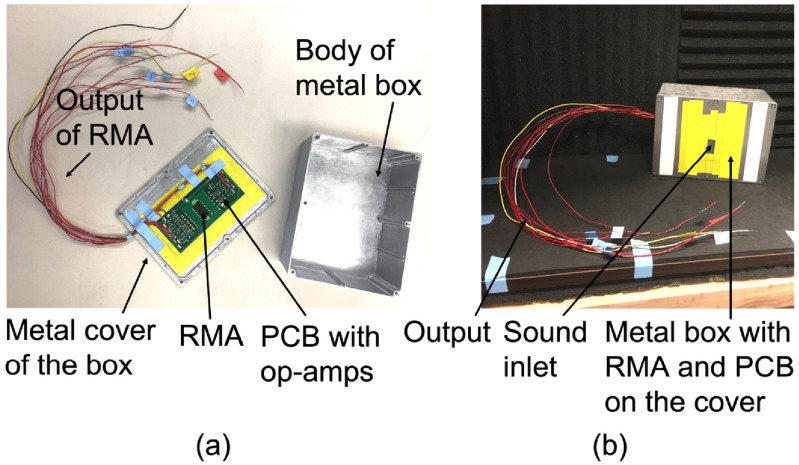
Photos of (a) the RMA on a PCB in a metal box and (b) the packaged RMA in an anechoic chamber.

**Figure 12.**
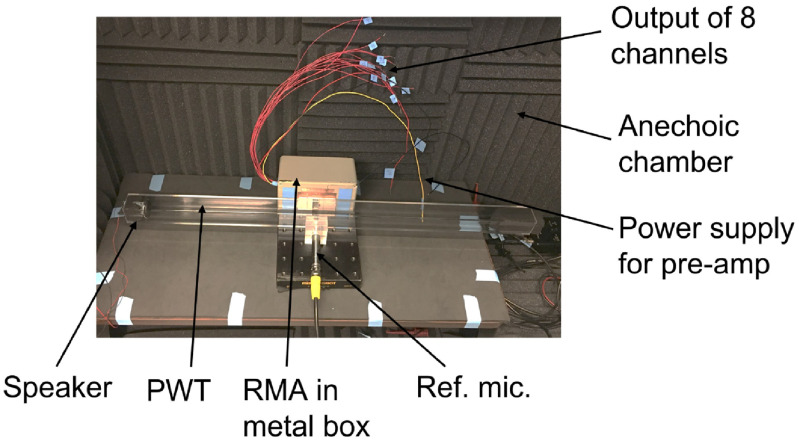
Photo of the microphone sensitivity measurement setup with a reference microphone GRAS 40AO across a plane wave tube (PWT), the cross section of which is $25.4\; \times \;25.4\;{\text{ m}}{{\text{m}}^2}$, in an anechoic chamber.

The PCB is mounted to a cover plate (with a slot for sound input) of a metal box which blocks electromagnetic interference (figure 11). The outputs of the microphone amplifiers are connected to a data acquisition system (ROGA Plug.n.DAQ). The sound input to the resonant microphone is calibrated with a reference measurement microphone (GRAS 40AO, noise floor 25 dBA, sensitivity 12.5 mV Pa^−1^, bandwidth 3.15 Hz–20 kHz) with both the RMA and the reference microphone being placed next to each other in a plane wave tube (PWT) in an anechoic chamber (figure 12). A loudspeaker placed at one end of the PWT delivers same sound pressure to the RMA and the reference microphone which are located near to each other.

**Figure 13.**
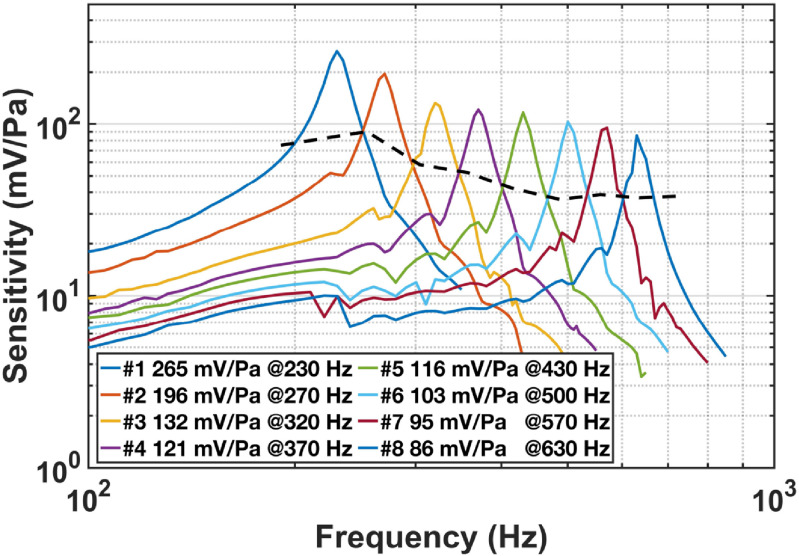
Measured unamplified sensitivities of the eight resonant microphones in the RMA. It is above 35 mV Pa^−1^ for the whole RMA between 200 Hz and 650 Hz as indicated by the black dash line.

**Figure 14.**
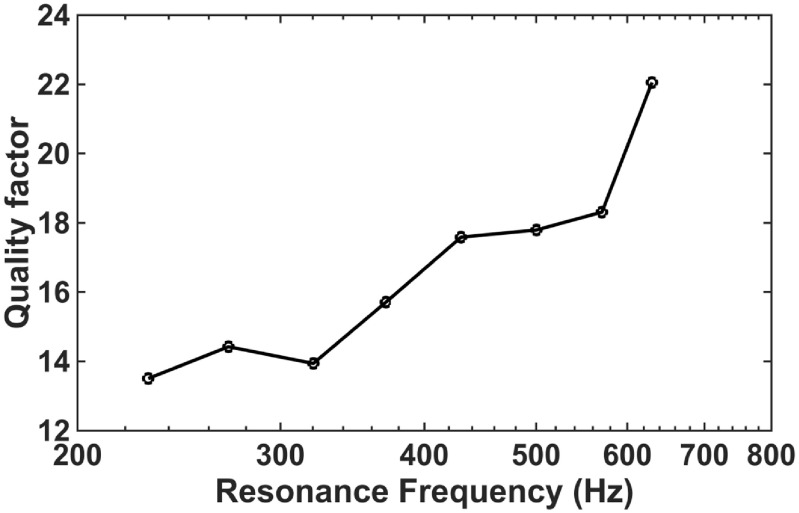
Measured quality factors of the resonant microphones in the RMA.

The measured unamplified sensitivities of the eight resonant microphones in the RMA are as high as 265–86 mV Pa^−1^ at the eight resonance frequencies (figure 13). The sensitivity of the RMA at all frequencies between 200 and 650 Hz is above 35 mV Pa^−1^ (above the dash line in figure 13). The measured resonance frequencies are lower than the designed ones (table 1), mainly because the Si cantilevers turn out to be 4 *μ*m thick, rather than 5 *μ*m thick. The sensitivity curve of each resonant microphone shows ripples near the resonance frequencies of the other resonant microphones due to electrical crosstalk among the resonant microphones in the RMA. This phenomenon can be reduced through better grounding of the microphones and amplification circuits. The quality factors (based on resonant frequency *f*_0_ divided by the −3 dB bandwidth in figure 13) of the resonant microphones are between 13.5 and 22 (figure 14). The damping coefficient of a smaller resonant microphone is usually smaller because the damping is mainly from the air surrounding the cantilever. Consequently, the quality factor is usually higher for the resonant microphones with higher resonance frequencies and smaller size. Higher quality factor leads to higher unamplified sensitivity and lower noise floor of a resonant microphone while the bandwidth (over which the sensitivity is enhanced by the resonance) is narrower. Therefore, we need proper quality factors for the resonant microphones in the RMA so that the RMA has both high sensitivities to detect weak sound and enough bandwidth to cover the frequency range of interest.

### 4.2. Noise floor and SNR

**Figure 15.**
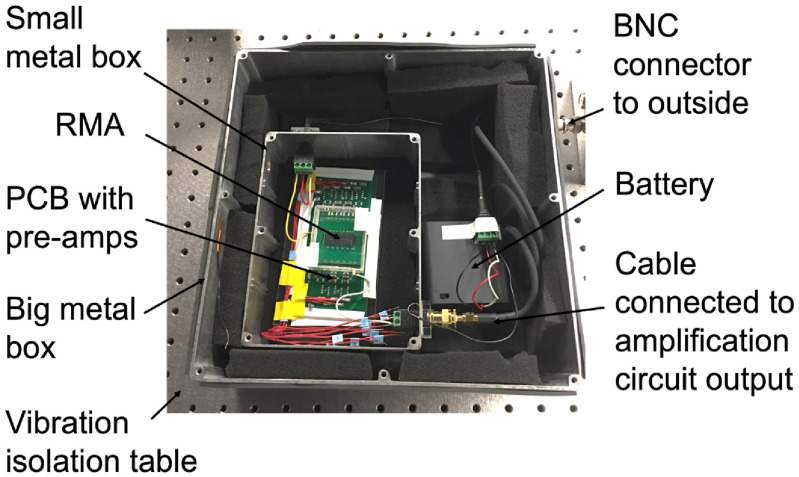
Photo of noise measurement setup with the RMA in a double metal box on an optical table for isolation from electromagnetic noise, acoustic noise, and mechanical vibration. The top of the metal box is covered during noise measurement.

To measure the noise without any electromagnetic or sound interference noise, the RMA and amplification circuit are placed in a double metal box with battery (figure 15).

**Figure 16.**
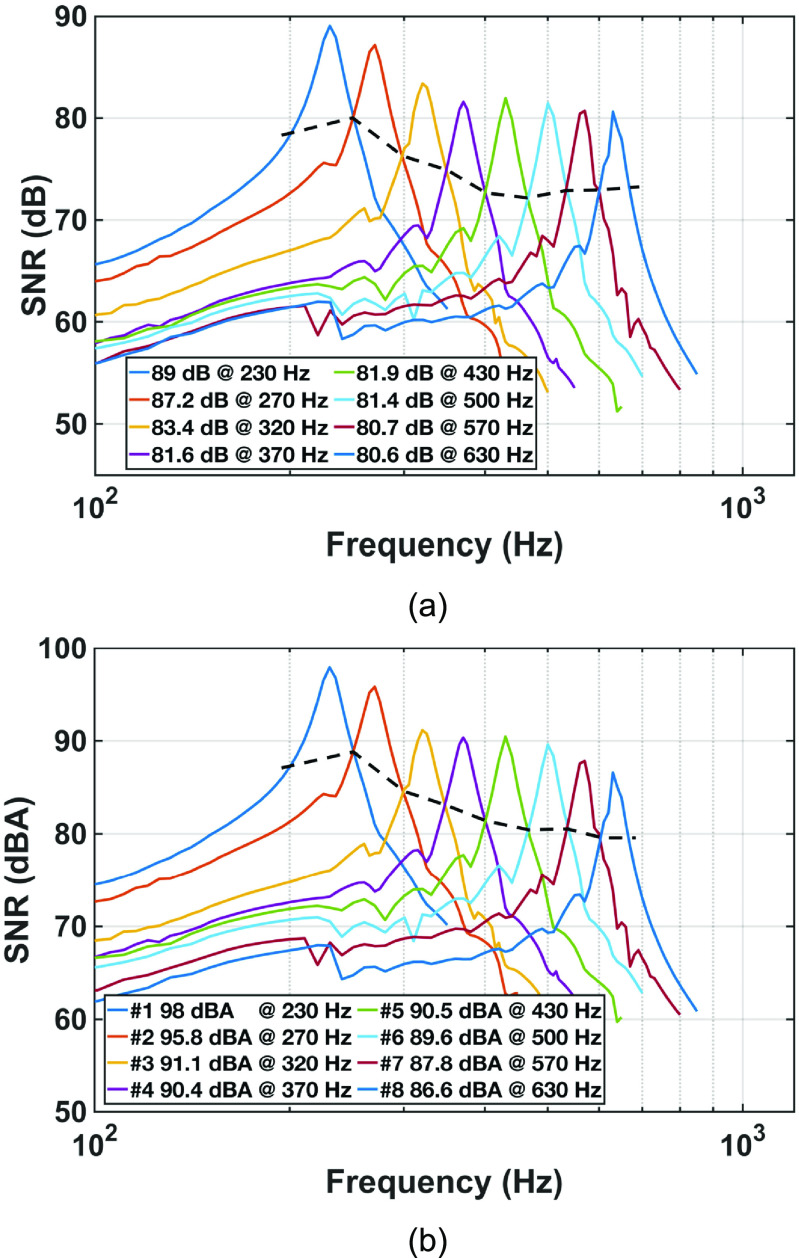
Measured signal-to-noise ratios (SNRs) of the resonant microphones in the RMA at 1 Pa sound pressure vs frequency (a) without A-weighting and (b) with A-weighting. The SNR of the whole array is above the black dash line.

**Figure 17.**
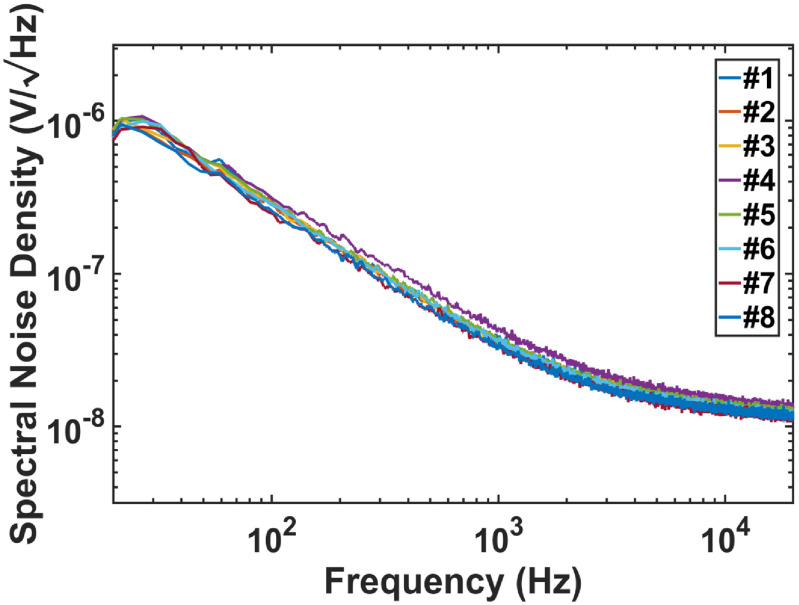
Measured noise spectral density of the resonant microphones in the RMA without A-weighting.

With the double metal box on a vibration isolation table, the output of the amplification circuit for each resonant microphone in the RMA is divided by the amplification (101) for input-referred noise of each resonant microphone and its amplifier. The measured input-referred root-mean-square (RMS) noise over 20 Hz–20 kHz observation bandwidth is 8–10 and 3–4 *μ*V before and after A-weighting, respectively. The noise floor in pressure is obtained by dividing the input-referred RMS noise voltage by the unamplified sensitivity, while the noise floor in dB is 20 log (noise-floor-in-Pa/reference-pressure) where the reference pressure is 2 × 10^−5^ Pa. And the SNR for 1 Pa sound pressure input is obtained by deducting the noise floor in dB from 94 dB, as 1 Pa sound pressure is 94 dB (= 20log(1/(2 × 10^−5^))). The measured SNRs of the resonant microphones at their resonances for 1 Pa sound pressure are 89.0–80.6 dB before A-weighting and 98.0–86.6 dBA after A-weighting (figure 16). Over all the frequencies between 200 and 650 Hz where wheezing in lung sounds is prominent, the SNR are 89.0–73.0 dB before A-weighting and 98.0–79.0 dBA after A-weighting (above the black dash line in figure 16). The spectral noise density of all resonant microphones in the RMA without A-weighting are lower than $1\,\mu V/\sqrt {Hz} $ (figure 17) with the $1/f$ noise of the op amp dominating, indicating that the external sound and vibration are isolated very well during the noise measurement.

## 5. Lung sounds detection and classification

Well-annotated lung sounds from International Conference on Biomedical and Health Informatics (ICBHI) Respiratory Sound Database [27] are played by a loudspeaker and recorded with the RMA and the reference microphone in a set-up shown in figure 12. The recordings are analyzed in time and frequency domains, and processed through deep learning and machine learning algorithms for automatic classification of wheezing in the lung sounds, to show the advantages of the RMA over a standard microphone.

### 5.1. Recorded signals in time and frequency domain

**Figure 18.**
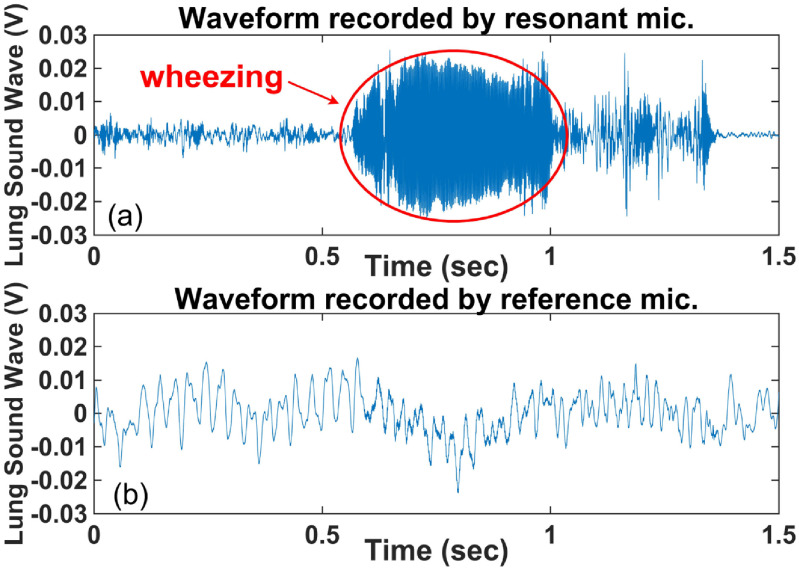
Waveform of a lung sound with strong wheezing recorded by (a) the resonant microphone #7 in the RMA (which records distinguishable wheezing) and (b) the reference microphone GRAS 40AO (which does not record distinguishable wheezing).

**Figure 19.**
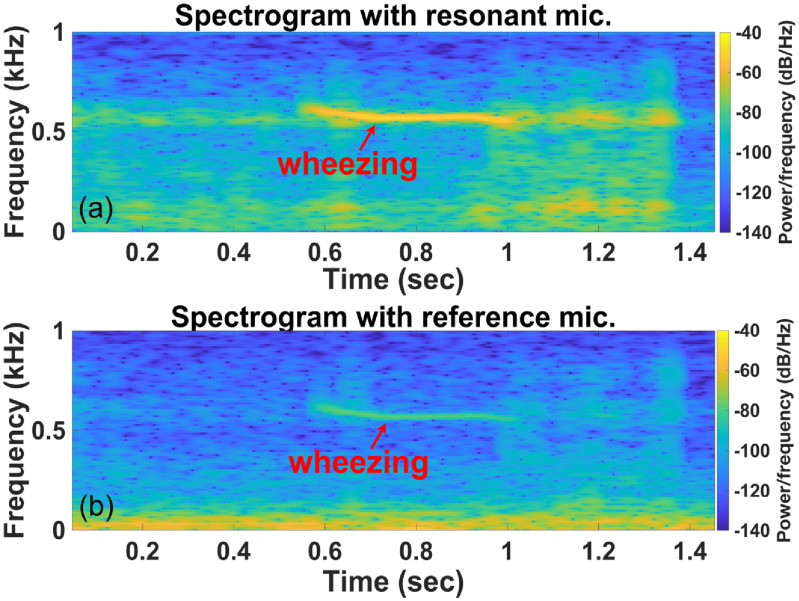
Spectrogram of a lung sound with strong wheezing recorded by (a) the resonant microphone #7 in the RMA (wheezing is distinguishable) and (b) the reference microphone (wheezing is distinguishable but not as obvious as the one recorded by the resonant microphone).

**Figure 20.**
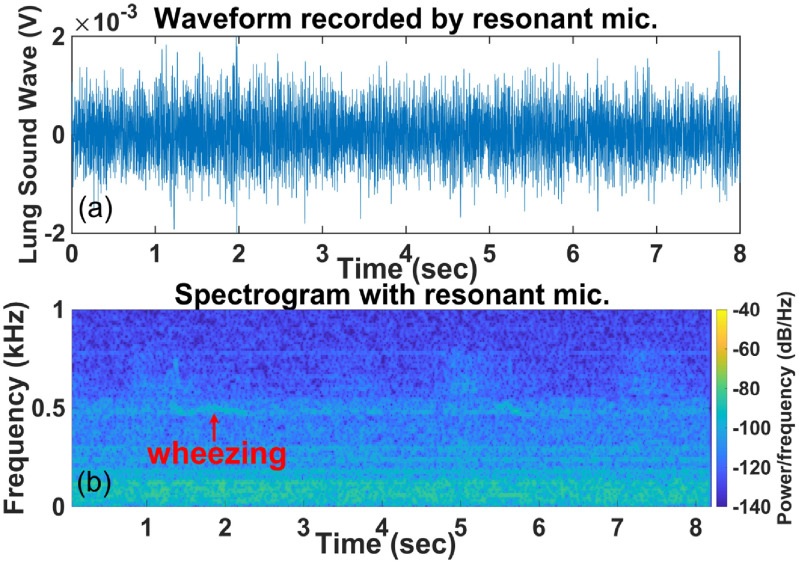
Waveform (a) and spectrogram (b) of a lung sound with weak wheezing recorded by the resonant microphone #6 in the RMA vs time; wheezing is not distinguishable in the waveform, while wheezing is distinguishable in the spectrogram.

**Figure 21.**
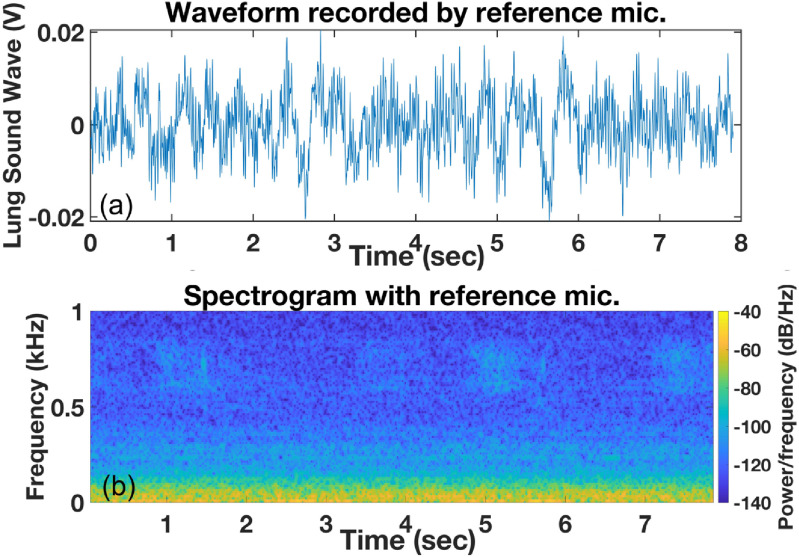
Waveform (a) and spectrogram (b) of a lung sound with weak wheezing recorded by the reference microphone; wheezing is not distinguishable.

Wheezing in lung sounds is easily recognizable in the recording by the RMA in time (figure 18(a)) and spectrogram (figure 19(a)), while the recording by the reference microphone shows little wheezing feature in time (figure 18(b)) and a weak feature in the spectrogram (figure 19(b)). A weak wheezing is not visible in the time recordings by the RMA and the reference microphone (figures 20(a) and 21(a)). Such a weak wheezing, though, can still be distinguished in the spectrogram of the recording by the RMA (figure 20(b)), but is not distinguishable in the spectrogram of the recording by the reference microphone (figure 21(b)). Thus, wheezing in the lung sounds can be recognized better in both time and frequency domain by the RMA than that by the conventional microphone.

### 5.2. Automatic wheeze classification with deep learning

Fifty lung sounds from the ICBHI database [27], with twenty-five of them having wheezing, are played by a loudspeaker and recorded by the RMA and the reference microphone (figure 12). The recordings are classified by deep learning algorithms, and the classification accuracies are compared.

With temporal convolutional networks (TCNs) [28], the recorded lung sounds in time domain are processed without any pre-processing for the classification. Twelve-layer networks are used in TCN to extract a ten-dimensioned feature vector for the classification. On the other hand, for convolutional neural networks (CNNs), pre-extracted Mel-frequency cepstral coefficients are used.

**Figure 22.**
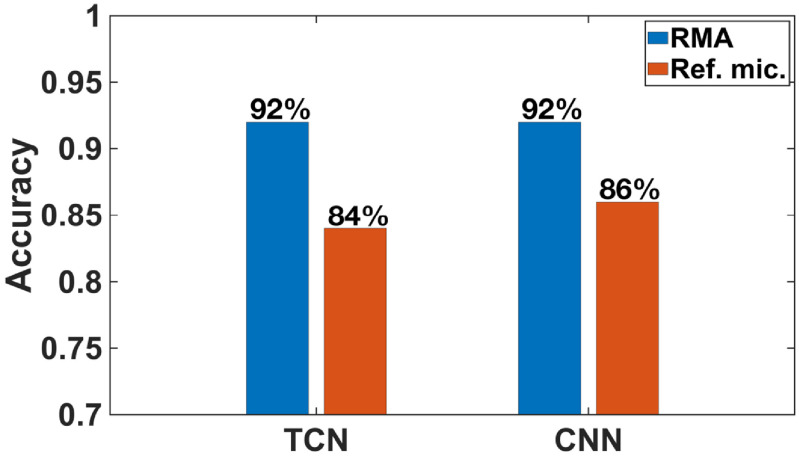
Wheezing automatic classification accuracy for lung sounds recorded by the RMA and the reference microphone and processed by two deep learning algorithms TCN and CNN.

K-fold cross-validation is applied so that all the data can be used for both training and test. The recordings are divided into *K* (5 in this case) groups randomly. One group is used for the test while the other groups are used for the training at each iteration until every group has been tested. The classification accuracy is the average accuracy of all the *K* iterations. With *K* being 5, 40 recordings are used for the training, and 10 recordings are used for the test in each iteration, for a total of 5 iterations. The classification accuracies of the lung sounds recorded by the RMA with both TCN and CNN are higher than what are obtained with the reference microphone (figure 22).

### 5.3. Automatic classification with machine learning on a chip set for wearable wireless communication

**Figure 23.**
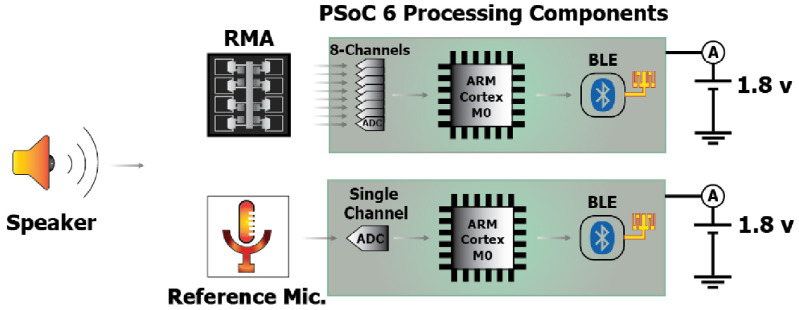
Schematic of the setups for audio recordings (by the RMA and the reference microphone) and processing with the wireless chip set (for feature extraction, classification through machine learning, and Bluetooth communication) to compare the performances of the RMA (top) and the reference microphone (bottom) for wheezing classification in lung sounds.

As deep learning algorithms cannot be implemented on a low power chip set for wireless communication such as Infineon CYBLE-416045-02 [29], which contains a microcontroller unit (MCU) and other components including antenna. We have developed and tested machine learning algorithms on the MCU (PSoC 63), which contains analog-to-digital converters, central processing unit, memory and blue tooth low energy transceiver (figure 23).

**Figure 24.**
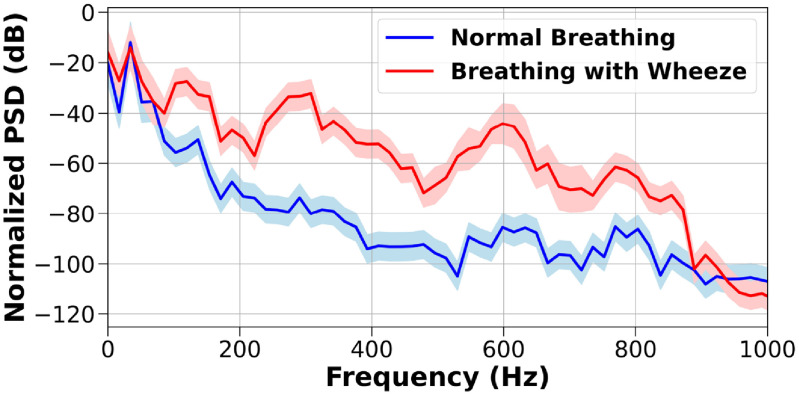
Spectral signature averaged per frame for lung sounds with and without wheezing recorded. The shaded regions indicate the standard deviation at each frequency. The power spectral density (PSD) of the lung sounds with wheezing is higher than that without wheezing especially between 300 and 600 Hz.

**Figure 25.**
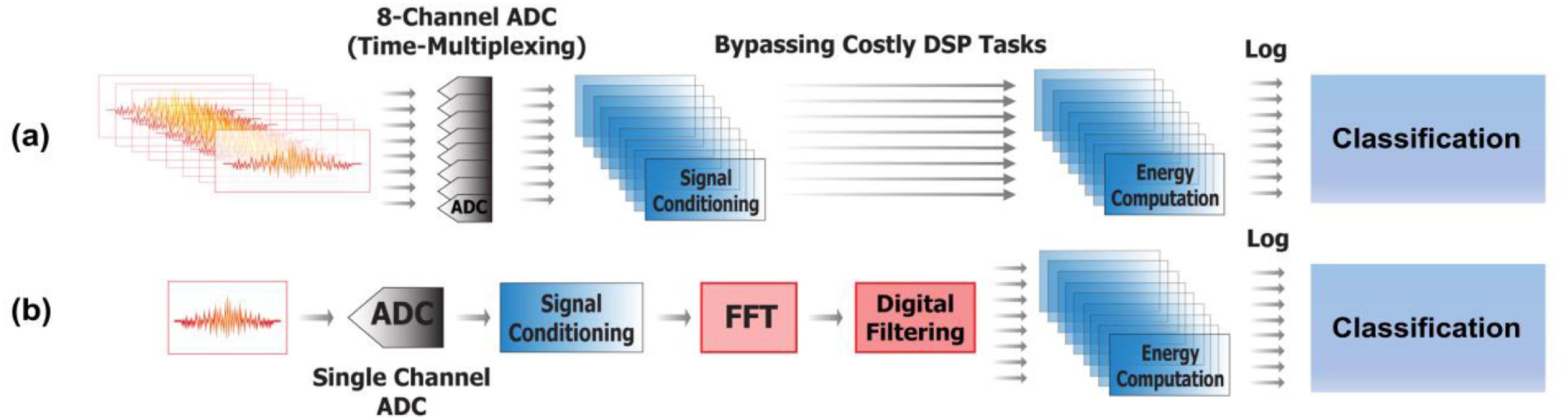
Block diagram illustrating the steps for the computing Mel-spectrogram features with (a) the eight-channel RMA and (b) the conventional approach using a traditional microphone with a digital filter bank. The FFT and multiple digital triangular filtering in (b) is not needed for (a).

**Figure 26.**
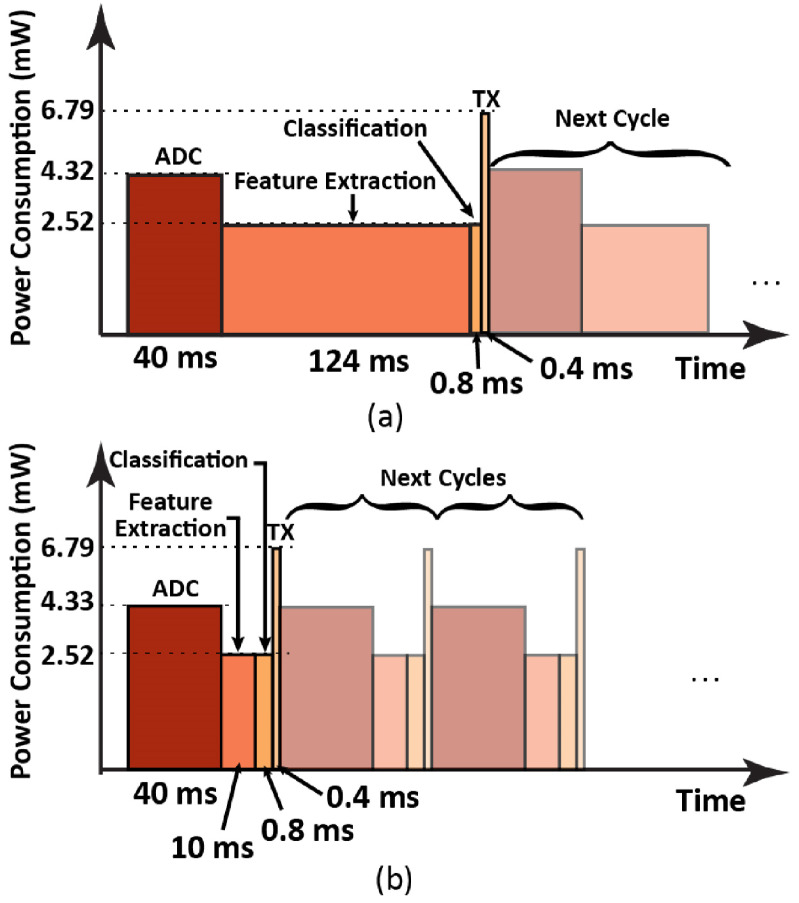
Power consumption vs time for sequential steps of automatic wheezing detection (a) with the reference microphone and (b) with the RMA. The feature extraction process by the RMA is ∼12 times faster and consumes 92% energy less than that by the reference microphone.

Each of the recordings is divided into 7650 pieces with each piece being 40 ms long for feature extraction. Mel-Spectrum features are extracted for classification as the frequency spectra of the lung sounds with and without wheezing are quite different (figure 24). On the data recorded by the reference microphone, fast Fourier transform (FFT) and digital filtering are applied for the feature extraction (figure 25(b)). However, with the data recorded by the RMA, the features at different frequencies are obtained through calculating the energy at each recording by individual resonant microphone in the RMA with its unique capability of acoustically filtering the audio signal (figure 25(a)). Thus, the feature extraction with the RMA is much faster (more than ten times) than that with the reference microphone as FFT is time consuming (figure 26). Though the idea of calculating the energy from individual channel of an RMA without FFT has been reported [30], the stronger MCU in PSoC 63 and optimized algorithms have resulted much faster signal processing.

**Figure 27.**
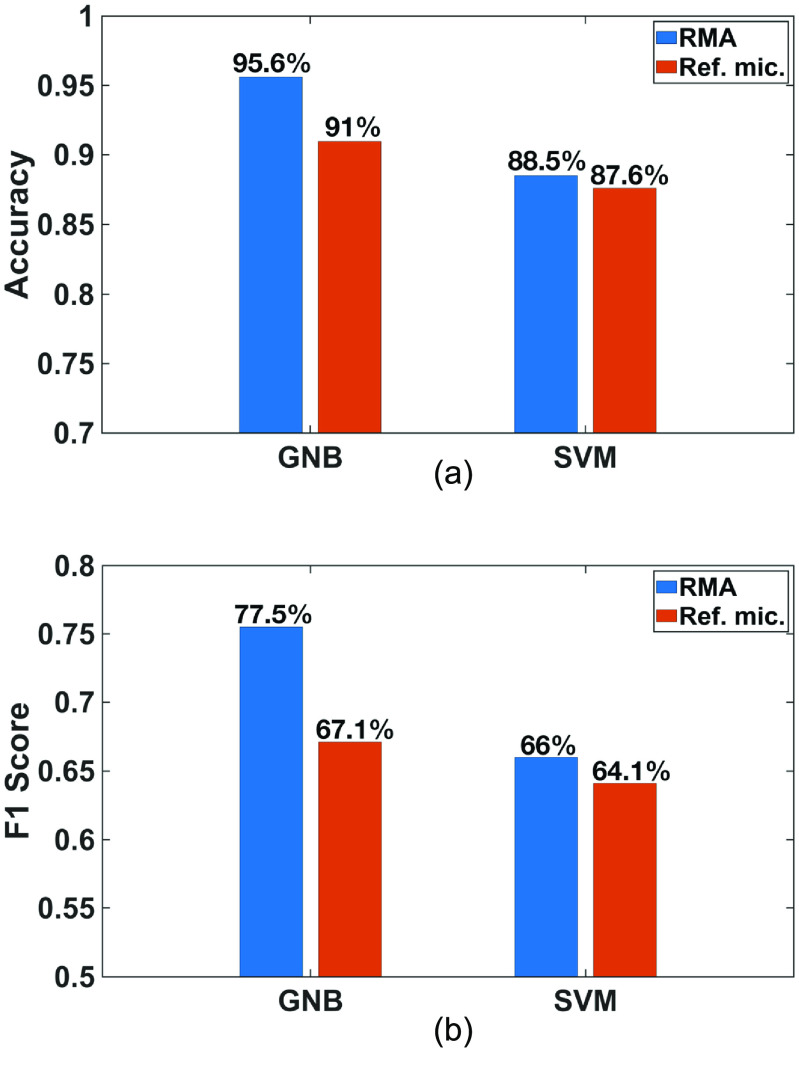
(a) Accuracies and (b) F1 scores of the classifications based on Gaussian Naïve Bayes (GNB) and support vector machine (SVM) algorithms for lung sounds recorded by the RMA and the reference microphone.

Two machine models, Gaussian Naïve Bayes and support vector machine, are developed for the classification based on the extracted features. These classifiers are trained for single frame prediction at each moment because temporal-variation and multi-frame analyses require more memory and processing speed from PSoC 63. The recorded data are split with 70% for training and 30% for testing. The training is implemented on a desktop computer, and then the parameters of the algorithms obtained from the training are transferred to PSoC 63 for the test. We have measured the classification accuracy which is equal to $\left( {{t_p} + {t_n}} \right)/\left( {{t_p} + {f_p} + \,{t_n} + {f_n}} \right)$ and F1 score which is equal to $2PR/\left( {P + R} \right)$, with ${t_{\text{p}}} \equiv $ true positive, ${t_{\text{n}}} \equiv $ true negative, ${f_{\text{p}}} \equiv $ false positive, ${f_{\text{n}}} \equiv $ false negative, $P \equiv {t_{\text{p}}}/\left( {{t_{\text{p}}} + {f_{\text{p}}}} \right)$ and $R \equiv {t_{\text{p}}}/\left( {{t_{\text{p}}} + {f_{\text{n}}}} \right)$. Both the classification accuracy and F1 score are better with the recordings with RMA than with the reference microphone (figure 27).

## 6. Discussion

**Figure 28.**
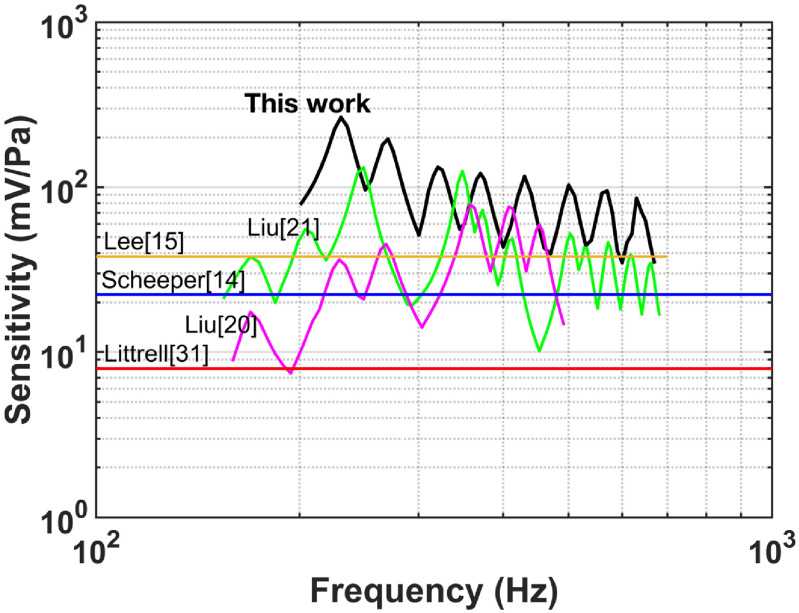
Comparison of the unamplified sensitivities of the reported MEMS microphones vs frequency.

**Figure 29.**
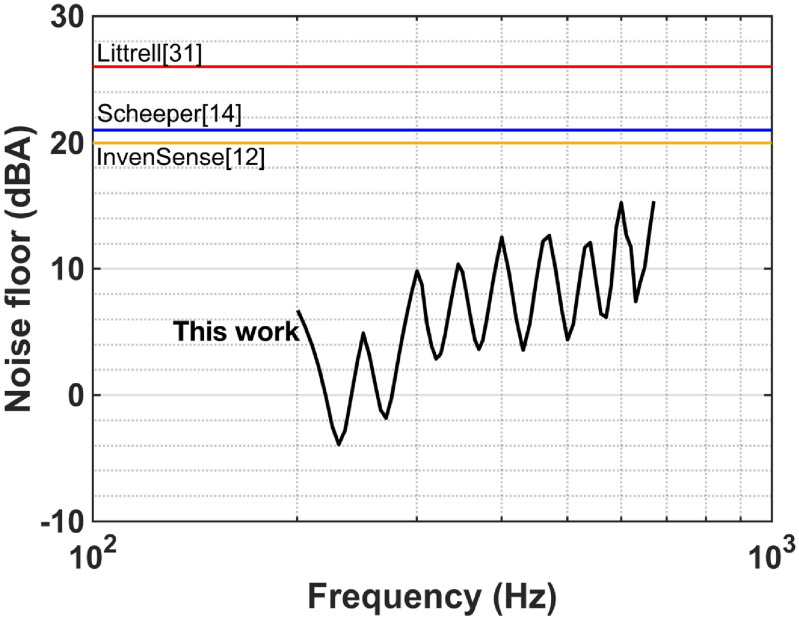
Comparison of the noise floors of the reported MEMS microphones vs frequency.

The unamplified sensitivities of the RMA (265–35 mV Pa^−1^) over 200–650 Hz where wheezing is prominent are higher than other MEMS microphones reported (figure 28), albeit with a larger size. The noise floor of the RMA also is lower than other reported MEMS microphones over 200–650 Hz (figure 29). Thus, the minimum detectable wheezing signature in lung sounds is better with the RMA. If a microphone is targeted for a limited frequency range, an RMA with multiple resonances over the frequency range is shown to offer unprecedented minimum detectable sound level. Although the sensitivity and noise floor of the RMA is not flat, we did not find the effect of this un-flatness on the lung sound classification.

Design innovation and optimization are important to make the resonant microphones small and highly sensitive. The width-stepped cantilever design with two narrow beams supporting a rectangular plate is shown to be smaller and to offer higher sensitivity than a standard cantilever (figures 2–4) which has much less bending stiffness than a diaphragm with its four edges clamped. The size can be reduced further with a spiral structure or cantilever with serpentine support beams [20, 21], which has exhibited less sensitivity than the current design in this paper.

**Figure 30.**
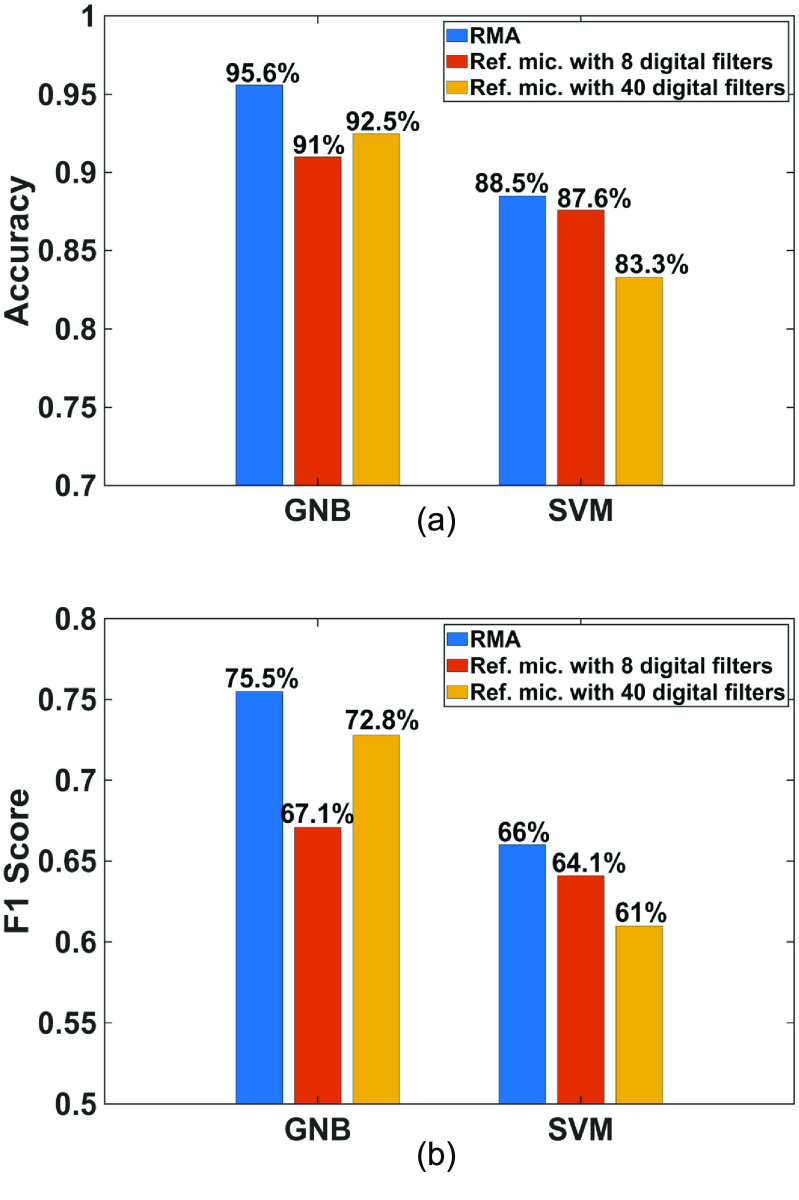
(a) Accuracies and (b) F1 scores for automatic classification of wheezing for lung sounds with the RMA without digital filters and the reference microphone with 8 and 40 digital filters.

With the conventional approach, the number of digital filters can be increased to improve the classification accuracy (figure 30), but at increased process time and power consumption. The accuracy and F1 score with reference microphone plus 40 filters are still lower than that with the proposed RMA. Therefore, more advanced algorithms with poor quality data from microphones with higher noise floor may not compete with simple algorithms with high quality data from the RMA with extremely low noise floor. Furthermore, the signal processing is much faster (figure 26) with the RMA which inherently filters sounds into specific bandwidths. Thus, the current work shows the significant advantages of the RMA for real-time lung sound monitoring and classification with a wearable stethoscope.

## 7. Summary

An array of piezoelectric MEMS resonant microphones, with novel width-stepped cantilever design with two narrow beams supporting a rectangular plate, has been developed with Mel-distributed resonance frequencies to cover the frequency range where wheezing in lung sounds is prominent, and is shown to have the highest unamplified sensitivity and SNR in this frequency range compared with other reported MEMS microphones. With the array, wheezing in lung sound is shown to be detected and automatically classified better than with a reference microphone. The automatic classification accuracies for wheezing are higher with the RMA for both deep learning (performed on a computer) and machine learning (performed on a chip set for wearable wireless communication). In addition, the signal processing with the RMA is shown to be more than ten times faster and consumes 92% less energy than that with a traditional microphone on a low power chip set for wearable wireless communication. Therefore, the current work paves the way for a wearable stethoscope to continuously monitor and automatically classify lung sounds in real-time so that patients or caregivers may be alerted and also so that medical professionals may have recordings of relevant lung sounds.

## Data Availability

All data that support the findings of this study are included within the article (and any supplementary files).
